# Exploring Postoperative Outcomes of Primary Repair Versus Loop Ileostomy in Typhoid Ileal Perforation: A Systematic Review and Meta-Analysis

**DOI:** 10.7759/cureus.89947

**Published:** 2025-08-12

**Authors:** Oluwatosin G Afolabi, Ajibola A Adebisi, Ebuka L Anyamene, David-Daniel Opone, Eniola Akande, Abdulahi Zubair

**Affiliations:** 1 General Surgery, Surgery Interest Group of Africa, Lagos, NGA; 2 General Surgery, Epsom and St Helier University Hospitals NHS Trust, London, GBR; 3 Clinical Sciences, University of Ilorin, Ilorin, NGA; 4 Neurosurgery, Surgery Interest Group of Africa, Lagos, NGA

**Keywords:** loop ileostomy, postoperative outcomes, primary repair, systematic review and meta-analysis, typhoid ileal perforation

## Abstract

Typhoid ileal perforation, a severe complication of typhoid fever, often leads to peritonitis and high mortality, particularly in low-income countries. While conservative management was once standard, surgical interventions like primary repair and loop ileostomy have been preferred since the 1970s, though outcomes vary due to late presentations and resource constraints. This systematic review and meta-analysis, adhering to Preferred Reporting Items for Systematic Reviews and Meta-Analyses (PRISMA) guidelines, evaluates postoperative morbidity, mortality, and hospital stay for these techniques. A comprehensive search of PubMed, Google Scholar, Cochrane Library, and African Journals Online from database inception to May 2025 identified 2,157 articles. After removing 1,215 duplicates and excluding 851 articles during title and abstract screening, 91 articles were sought for retrieval. Full texts of 24 studies were unavailable. Thirty-eight articles were excluded after full-text screening of 67 studies, leaving 29 peer-reviewed studies comparing primary repair and ileostomy outcomes for typhoid ileal perforation. The analysis included 2,434 patients from five countries (Côte d'Ivoire, India, Nigeria, Pakistan, and Turkey, with India and Pakistan contributing the most). Of these, 1,315 underwent primary repair, and 1,119 had ileostomy. Study designs comprised comparative, randomized controlled, retrospective, prospective, observational, and quasi-experimental studies. Participants had a mean age of 28.04 years (SD=9.39), with a male predominance (62.9% male vs. 25.1% female). The meta-analysis of 23 studies showed that ileostomy is associated with an 18.4% higher postoperative complication rate compared to primary repair. For mortality, analysis of 22 studies indicated a 3.1% higher rate with ileostomy, though this difference was not significant, with variability suggesting influences beyond procedure type, such as patient condition and intraoperative findings. Hospital stay analysis from 12 studies revealed that ileostomy patients stayed approximately 1.8 days longer than those undergoing primary repair, with considerable variation likely due to complications, stoma management practices, and institutional discharge protocols. These findings highlight the need for individualized surgical decision-making based on intraoperative findings, patient physiology, and resource constraints, especially in low-resource settings where typhoid perforation is prevalent.

## Introduction and background

Typhoid fever, also known as enteric fever, is a severe febrile illness caused primarily by the gram-negative bacterium *Salmonella typhi*. It remains a major public health problem in developing countries, affecting 13-17 million people internationally each year [1,2]. The emergence of multidrug-resistant strains has led to high incidences of morbidity and mortality, particularly in remote areas with limited medical facilities [3]. One of the most dreaded and common complications of typhoid fever is intestinal perforation, which frequently occurs in the terminal ileum during the second or third week of illness and often results in diffuse peritonitis [4-6]. This complication continues to be a leading cause of high morbidity and mortality, especially in developing countries where mortality rates range from 9% to 22%, compared to 0-2% in developed regions [7].

Historically, typhoid intestinal perforation was considered almost fatal, with conservative management being the norm until the 1960s [8]. However, since the 1970s, surgical intervention has become the preferred approach [9]. The management of this condition poses unique challenges to surgeons due to late presentation, often exacerbated by factors such as delayed diagnosis, initial treatment by unqualified practitioners, injudicious use of steroids, poverty, lack of transport, and poor medical infrastructure [10]. Current surgical options include layered closure, segmental resection with end-to-end anastomosis, and primary ileostomy [11-13]. Among these, primary repair and loop ileostomy are widely debated techniques, with varying outcomes reported in the literature [14]. Despite remarkable improvements in surgical management, mortality and morbidity rates remain high, particularly in cases presenting late with heavy fecal contamination [15].

This study aims to explore postoperative outcomes of primary repair versus loop ileostomy in typhoid ileal perforation through a systematic review and meta-analysis, focusing on key outcomes including morbidity, mortality, and hospital stay. There have been numerous individual comparative studies evaluating these surgical techniques, but no systematic review or meta-analysis exists in the literature to synthesize this evidence. In this study, we sought to fill this gap by comparing outcomes across available data and reviewing the associated complications, thereby providing a comprehensive analysis to guide clinical practice. Early surgery is considered the best treatment to contain further peritoneal contamination, yet no single procedure has proven universally satisfactory due to the varying pros and cons of each approach [16]. Studies comparing different surgical techniques have yielded controversial results, leaving a difference of opinion regarding the optimal procedure [17]. By synthesizing existing evidence, this research seeks to provide clarity on the most effective surgical strategy for managing this life-threatening condition.

## Review

Methods

This systematic review was conducted in accordance with the Preferred Reporting Items for Systematic Reviews and Meta-Analyses (PRISMA) extension for systematic reviews [18]. The study protocol was registered with the International Prospective Register of Systematic Reviews (PROSPERO) (CRD420251053737).

Eligibility Criteria

The eligibility criteria for the systematic review and meta-analysis were defined based on the PICO (Population, Intervention, Comparison, and Outcome) framework as follows:

Inclusion criteria: Included were primary studies published in peer-reviewed journals involving patients of any age or gender diagnosed with typhoid ileal perforation, confirmed through clinical, radiological, or microbiological evidence, in both hospital and community settings across various geographical locations and socioeconomic backgrounds, requiring surgical intervention. These studies must evaluate primary repair of the perforation, using techniques such as single-layer or two-layer closure, with or without an omentum patch, as the primary procedure and compare primary repair with loop ileostomy, which could be performed alone or combined with other techniques like resection, as an alternative surgical approach. The studies must report at least one postoperative outcome, including morbidity such as wound infection, anastomotic leak, or intra-abdominal abscess, mortality, for instance, within 30 days post-surgery or during hospital stay, and the duration of hospitalization in days, providing quantitative data like rates, means, or medians. The study designs included retrospective, prospective, comparative, quasi-experimental, and observational studies and randomized controlled trials, with all studies published in English.

Exclusion criteria: Excluded were studies involving patients with perforations caused by conditions other than typhoid, such as duodenal ulcer or small bowel tuberculosis, or those not requiring surgical intervention and also studies that did not evaluate primary repair as the surgical intervention or failed to compare primary repair directly with loop ileostomy, such as those focusing solely on other procedures like resection and anastomosis without reference to either primary repair or ileostomy. Additionally, studies were excluded if they did not provide quantitative data on postoperative outcomes, like morbidity, mortality, or hospital stay duration, or if they only offered qualitative or narrative outcomes without measurable data. The excluded study designs included case reports, case series, narrative reviews, systematic reviews, meta-analyses, editorials, conference abstracts, and letters to editors. Studies published in languages other than English were also excluded.

To identify all eligible articles, a comprehensive search was conducted from inception to May 30, 2025. PubMed, Google Scholar, Cochrane Library, and African Journals Online were searched to retrieve relevant studies. During the screening process, the references of similar review articles were manually searched to identify any studies that might have been missed in the initial search. The search strategy was collaboratively developed by the authors and is detailed in the Appendices. Duplication, title, and abstract screening were conducted by five independent reviewers (OGA, AA, ELA, DDO, and EA) using the Rayyan systematic review software (Rayyan Systems Inc., Cambridge, Massachusetts, United States), guided by predefined eligibility criteria. Potentially eligible studies underwent full-text review. Discrepancies among reviewers were addressed through discussion, with an additional reviewer (AZ) consulted if consensus could not be reached. Data extraction from the articles included details on the author, study year, sample size, study area, mean age, gender, morbidity rates, mortality rates, causes of mortality, and duration of hospital stay.

Comparative studies form the largest proportion of the included literature, highlighting the importance of side-by-side evaluations of primary repair versus ileostomy. The presence of randomized controlled trials, though few, strengthens the overall evidence base by contributing higher levels of clinical evidence. The methodological analysis of the quality of studies was conducted using several tools. For the eight retrospective, two observational, and one prospective studies, the Newcastle-Ottawa Scale (NOS) [19] was employed to assess the risk of bias. For the two randomized controlled trials, the Cochrane Risk of Bias (RoB) 2 tool [20] was used to evaluate bias, ensuring a thorough assessment of randomization, blinding, and outcome reporting. Additionally, the four quasi-experimental and 12 comparative studies were assessed using the Risk Of Bias In Non-randomized Studies - of Interventions (ROBINS-I) tool [21]. The results of the risk of bias assessment are provided in Table 1. 

**Table 1:** Risk of bias assessments NOS: Newcastle-Ottawa Scale; Cochrane RoB 2: Cochrane Risk of Bias 2; ROBINS-I: Risk Of Bias In Non-randomized Studies - of Interventions

Authors	Risk of bias tool	Score
Waqar and Khan [1]	Cochrane RoB 2 tool	Low risk
Osifo and Ogiemwonyi [3]	NOS	6
Kouame et al. [4]	NOS	8
Mittal et al. [16]	ROBINS-I tool	7
Ali et al. [22]	NOS	8
Noorani et al. [23]	NOS	8
Khan et al. [24]	NOS	7
Atamanalp et al. [25]	NOS	8
Mishra et al. [26]	NOS	7
Ugochukwu et al. [27]	NOS	7
Neelma et al. [28]	Cochrane RoB 2 tool	Low risk
Gul et al. [29]	ROBINS-I tool	8
Nsar et al. [30]	ROBINS-I tool	6
Farooq et al. [31]	ROBINS-I tool	6
Shah et al. [32]	ROBINS-I tool	7
Kumar et al. [33]	NOS	6
Ashrad et al. [34]	NOS	8
Rathod et al. [35]	NOS	6
Zardari et al. [36]	ROBINS-I tool	6
Khan et al. [37]	ROBINS-I tool	7
Shah et al. [38]	ROBINS-I tool	8
Ullah et al. [39]	ROBINS-I tool	8
Cheema et al. [40]	ROBINS-I tool	6
Beniwal et al. [41]	ROBINS-I tool	8
Kapoor et al. [42]	ROBINS-I tool	6
Thakre et al. [43]	ROBINS-I tool	6
Asif et al. [44]	ROBINS-I tool	7
Agrawal et al. [45]	ROBINS-I tool	6
Arshad et al. [46]	ROBINS-I tool	7

Results

**Figure 1:**
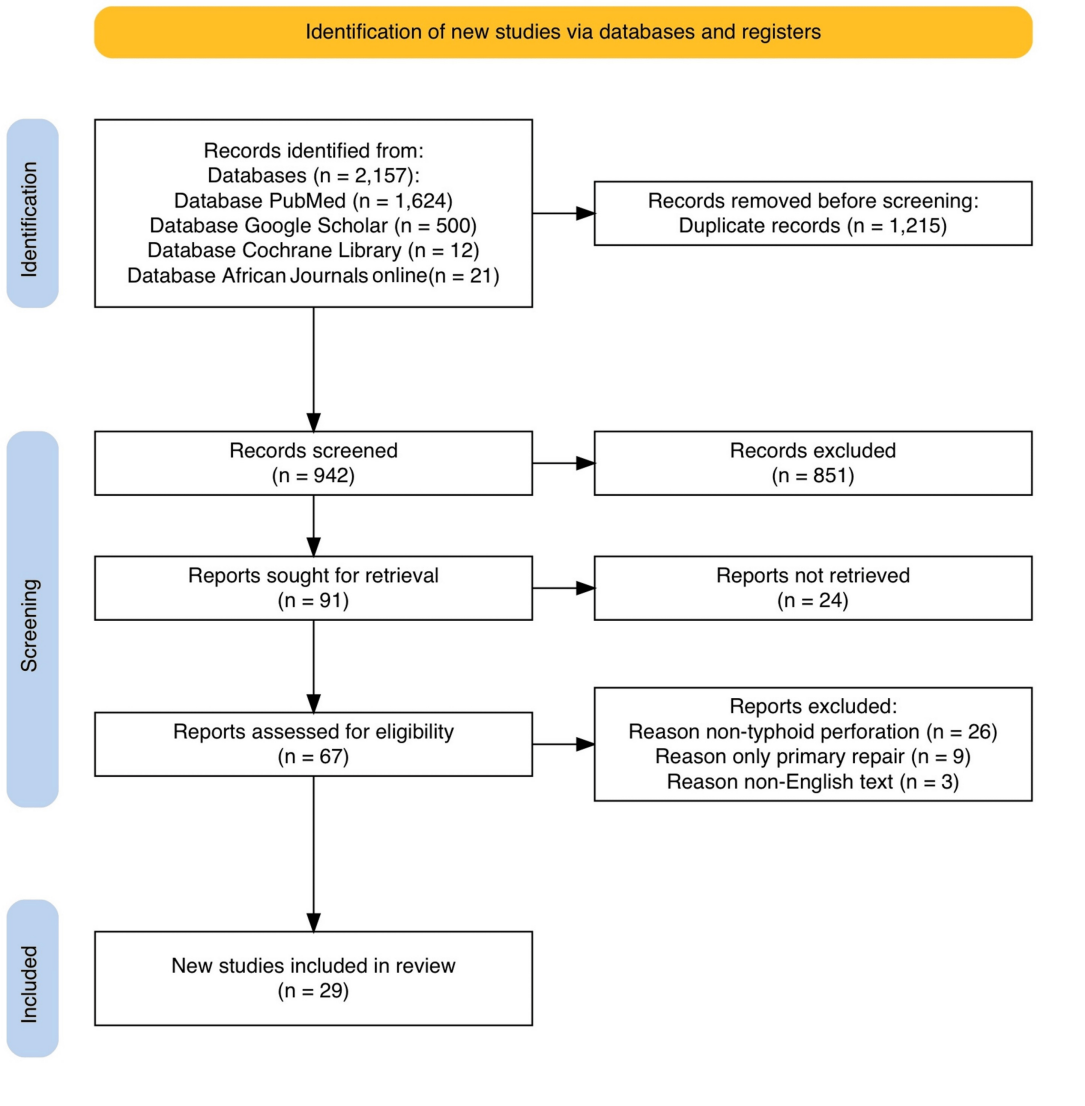
PRISMA flow diagram PRISMA: Preferred Reporting Items for Systematic Reviews and Meta-Analyses

Our search yielded 2,157 articles, of which 942 were screened by title and abstract after duplicates were removed. Following this initial screening, 851 articles were excluded, and while 61 were sought for retrieval, full texts of 24 could not be obtained. Of the remaining 67 articles subjected to full-text screening to assess eligibility against our inclusion criteria, 29 were ultimately included in the final qualitative synthesis. Figure 1 presents the PRISMA flow diagram. Exclusions were based on various reasons: 26 studies addressed non-typhoid perforations, nine focused solely on primary repair or other surgical techniques without an ileostomy as a comparator, failing to meet the inclusion criteria, and three were non-English texts. Data screening and extraction were performed independently by five authors, with a sixth reviewer consulted to resolve any disagreements.

*Sociodemographic Characteristics*

**Figure 2:**
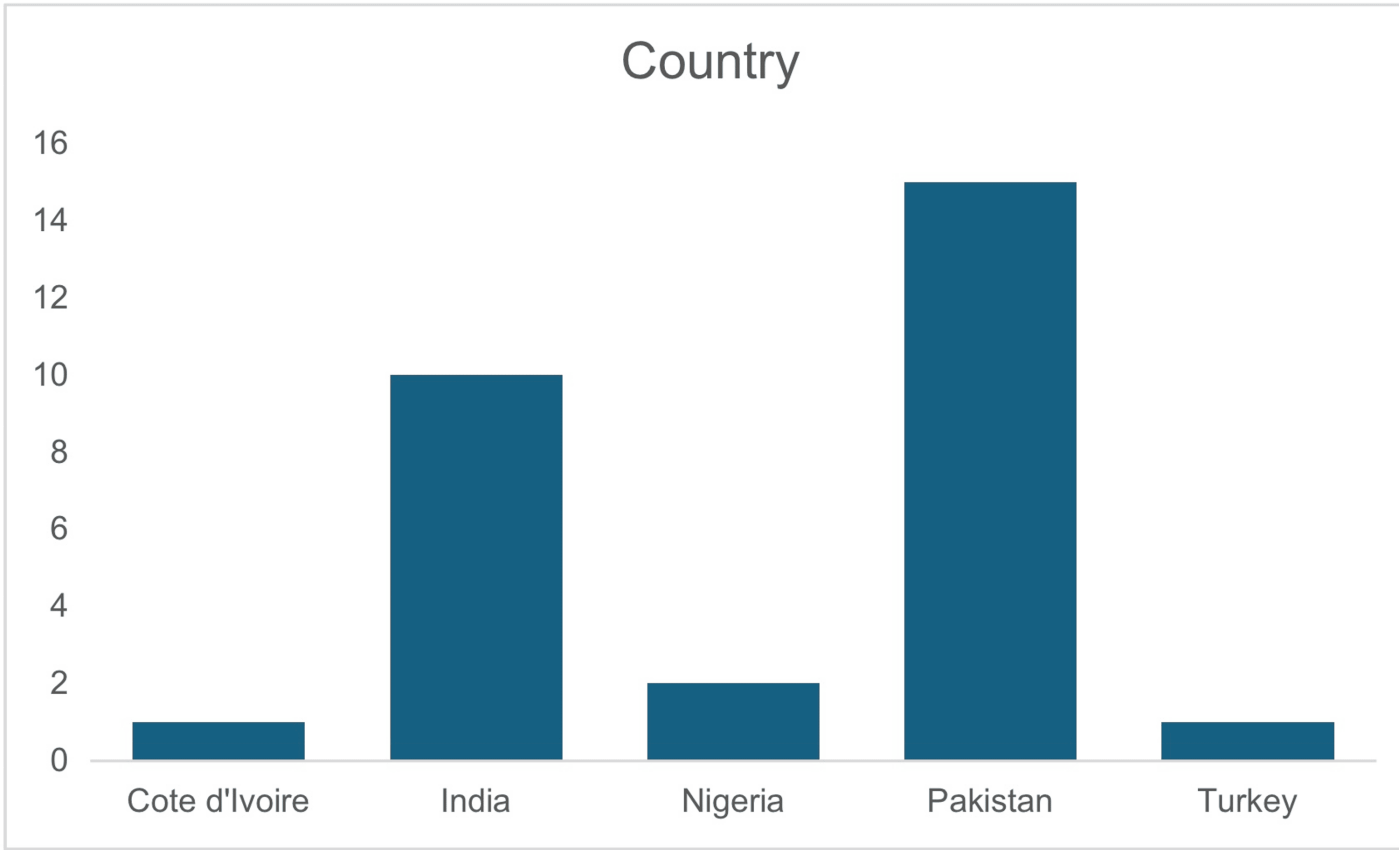
Geographic distribution of the studies

**Figure 3:**
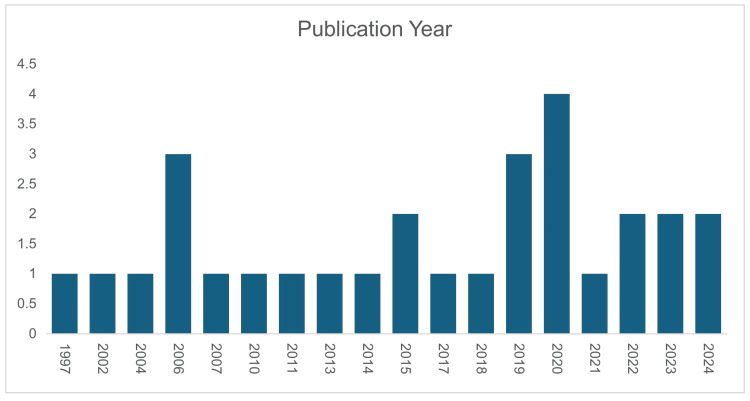
Publication year of the studies

**Figure 4:**
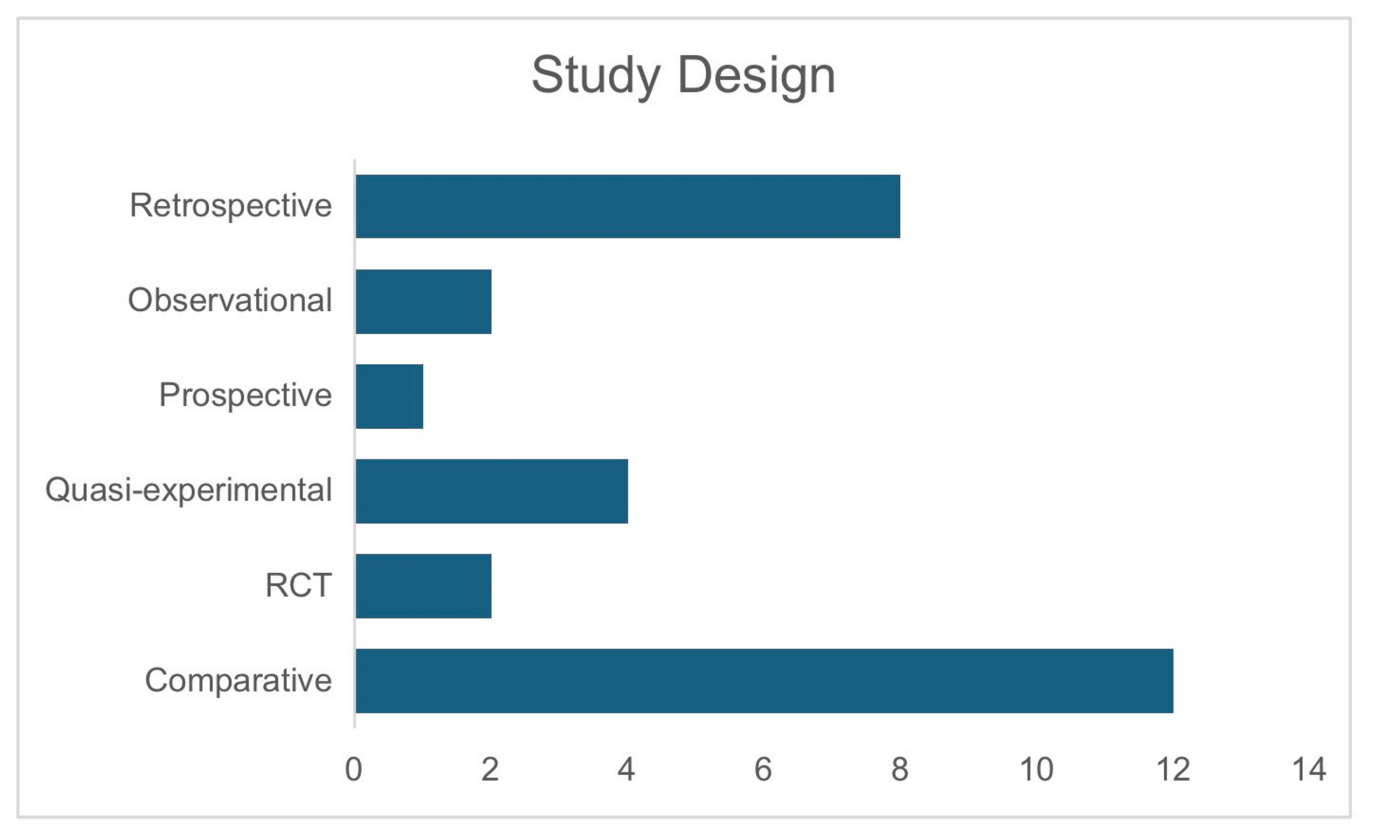
Study design RCT: randomized controlled trial

The meta-analysis included 2,434 patients across 29 studies from five countries (Nigeria, India, Pakistan, Côte d'Ivoire, and Turkey), with India and Pakistan contributing the majority of studies. Figure 2 illustrates the study locations. Of these patients, 1,315 underwent primary repair, and 1,119 had an ileostomy. Spanning from 1977 to 2024 (Figure 3), the studies focused primarily on low- and middle-income countries, where typhoid fever is most prevalent, ensuring sociodemographic relevance. The mean age of participants was 28.04 years (SD=9.39), indicating that typhoid intestinal perforation predominantly affects young adults, a demographic with significant socioeconomic impact (Figure 3). The sample showed a male predominance, with 62.9% males compared to 25.1% females. Study designs included retrospective [3,4,22-27], quasi-experimental [29-32], prospective [33], observational [34,35], and comparative studies [16,36-46] and randomized controlled trials [1,28] (Figure 4).

*Morbidity* 

**Figure 5:**
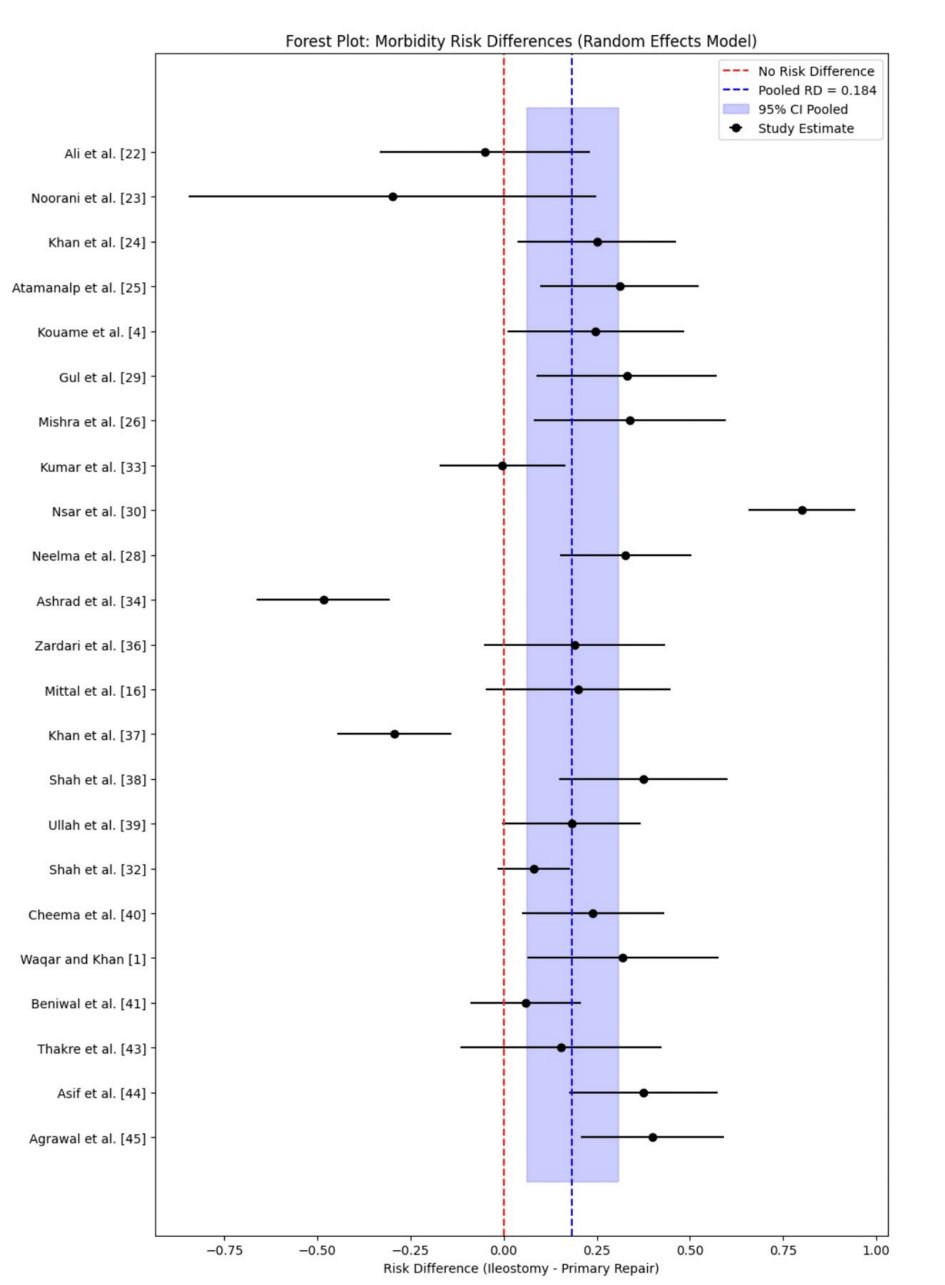
Forest plot of morbidity rate risk differences with pooled estimates

A meta-analysis comparing postoperative morbidity between primary repair and ileostomy for typhoid intestinal perforation revealed a pooled risk difference (RD) of 0.184 (18.4%), favoring primary repair. The 95% confidence interval (0.08-0.29) excludes zero, confirming a statistically significant increase in complications with ileostomy (Table 2). The forest plot (Figure 5) illustrates variability across individual studies, with most effect estimates positioned to the right of the null line, reinforcing higher morbidity in the ileostomy group. Despite inter-study heterogeneity, the random-effects model consistently indicates greater postoperative complications with ileostomy.

**Table 2:** Morbidity rates in primary repair vs. ileostomy RD: risk difference; SE: standard error; CI: confidence interval

Author(s)	Intervention (primary repair)	Intervention (ileostomy)	Morbidity (primary repair)	Morbidity (ileostomy)	RD	Variance difference	SE	Lower CI	Upper CI
Ali et al. [22]	20	30	11	15	-0.050	0.021	0.144	-0.332	0.232
Noorani et al. [23]	57	3	36	1	-0.298	0.078	0.280	-0.846	0.250
Khan et al. [24]	40	40	16	26	0.250	0.012	0.108	0.038	0.462
Atamanalp et al. [25]	41	32	18	24	0.311	0.012	0.109	0.097	0.524
Kouame et al. [4]	31	33	13	22	0.247	0.015	0.121	0.011	0.484
Osifo and Ogiemwonyi [3]	2	2	2	2	0.000	0.000	0.000	0.000	0.000
Gul et al. [29]	52	21	15	13	0.331	0.015	0.123	0.089	0.572
Mishra et al. [26]	41	19	12	12	0.339	0.017	0.132	0.081	0.597
Kumar et al. [33]	42	58	32	44	-0.003	0.007	0.086	-0.173	0.166
Nsar et al. [30]	30	30	6	30	0.800	0.005	0.073	0.657	0.943
Arshad et al. [46]	35	35	N/A	N/A	N/A	N/A	N/A	N/A	N/A
Neelma et al. [28]	55	55	17	35	0.327	0.008	0.090	0.151	0.504
Ugochukwu et al. [27]	52	7	N/A	N/A	N/A	N/A	N/A	N/A	N/A
Farooq et al. [31]	23	23	N/A	N/A	N/A	N/A	N/A	N/A	N/A
Ashrad et al. [34]	40	54	29	13	-0.484	0.008	0.091	-0.664	-0.305
Zardari et al. [36]	45	23	13	11	0.189	0.015	0.124	-0.054	0.433
Mittal et al. [16]	30	30	12	18	0.200	0.016	0.126	-0.048	0.448
Khan et al. [37]	75	75	45	23	-0.293	0.006	0.078	-0.446	-0.141
Shah et al. [38]	32	32	8	20	0.375	0.013	0.115	0.150	0.600
Ullah et al. [39]	51	52	26	36	0.183	0.009	0.095	-0.003	0.368
Shah et al. [32]	200	200	104	120	0.080	0.002	0.049	-0.017	0.177
Cheema et al. [40]	50	50	20	32	0.240	0.009	0.097	0.050	0.430
Waqar and Khan [1]	25	25	6	14	0.320	0.017	0.131	0.063	0.577
Beniwal et al. [41]	113	70	53	37	0.060	0.006	0.076	-0.089	0.208
Kapoor et al. [42]	25	25	N/A	N/A	N/A	N/A	N/A	N/A	N/A
Rathod et al. [35]	19	7	N/A	N/A	N/A	N/A	N/A	N/A	N/A
Thakre et al. [43]	24	26	12	17	0.154	0.019	0.138	-0.117	0.425
Asif et al. [44]	40	40	16	31	0.375	0.010	0.102	0.176	0.574
Agrawal et al. [45]	25	22	15	22	0.400	0.010	0.098	0.208	0.592

Disaggregated data further support these findings. Studies such as Mishra et al. [26] (RD=0.34), Cheema et al. [40] (RD=0.24), Atamanalp et al. [25] (RD=0.31), and Asif et al. [44] (RD=0.38) reported substantially higher morbidity in the ileostomy group, consistently favoring primary repair.

*Mortality* 

**Figure 6:**
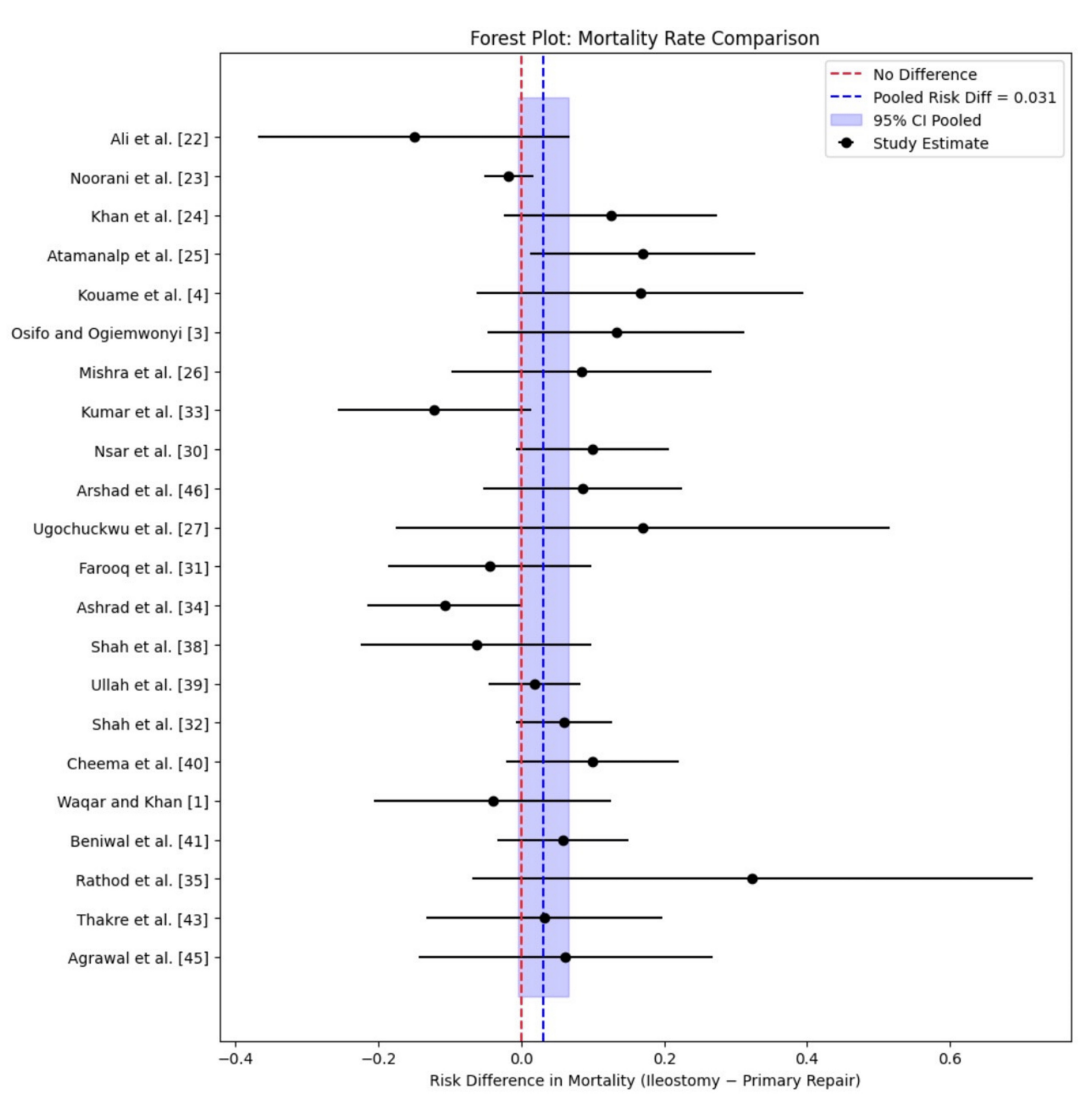
Forest plot of mortality rate risk differences with pooled estimates

The pooled meta-analysis of 22 studies reveals an RD of +0.031, suggesting that ileostomy is associated with a 3.1% higher mortality rate than primary repair (Table 3). However, the 95% confidence interval crosses zero, indicating that this difference is not statistically significant. The forest plot (Figure 6) displays considerable variability, with several studies showing risk differences both in favor of primary repair and ileostomy. This heterogeneity suggests that mortality outcomes are influenced by factors beyond the procedure type alone, such as timing of presentation, severity of contamination, and intraoperative findings, rather than a uniformly higher risk associated with ileostomy itself [1,21,23,29,38]. Several studies demonstrate elevated mortality rates in the ileostomy group, including Atamanalp et al. [25] (RD=0.17) and Kouame et al. [4] (RD=0.166). This may reflect selection bias, as ileostomy is often chosen in critically ill patients with multiple perforations, late presentation, or extensive peritoneal contamination [19,20,31]. Nonetheless, other studies report equal or even higher mortality in the primary repair group, such as Kumar et al. [33] (RD=-0.122) and Ashrad et al. [34] (RD=-0.106), almost always due to fecal fistula and sepsis. These findings emphasize that primary repair is not without risk, especially when performed in unstable patients or in centers with limited postoperative monitoring. Additionally, some studies like Ullah et al. [39] (RD=0.019) and Cheema et al. [40] (RD=0.100) report only marginal differences, reinforcing that mortality may be multifactorial [1,16,35].

**Table 3:** Mortality rates in primary repair vs. ileostomy RD: risk difference; SE: standard error; CI: confidence interval

Author(s)	Intervention (primary repair)	Intervention (ileostomy)	Mortality (primary repair)	Mortality (ileostomy)	RD	Variance difference	SE	Lower CI	Upper CI
Ali et al. [22]	20	30	5	3	-0.150	0.012	0.111	-0.368	0.068
Noorani et al. [23]	57	3	1	0	-0.018	0.000	0.017	-0.052	0.017
Khan et al. [24]	40	40	3	8	0.125	0.006	0.076	-0.023	0.273
Atamanalp et al. [25]	41	32	2	7	0.170	0.006	0.080	0.012	0.328
Kouame et al. [4]	31	33	8	14	0.166	0.014	0.117	-0.062	0.395
Osifo and Ogiemwonyi [3]	2	2	2	N/A	N/A	N/A	N/A	N/A	N/A
Gul et al. [29]	52	21	3	4	0.133	0.008	0.092	-0.047	0.312
Mishra et al. [26]	41	19	3	3	0.085	0.009	0.093	-0.098	0.267
Kumar et al. [33]	42	58	8	4	-0.122	0.005	0.069	-0.257	0.014
Nsar et al. [30]	30	30	0	3	0.100	0.003	0.055	-0.007	0.207
Arshad et al. [46]	35	35	2	5	0.086	0.005	0.071	-0.053	0.225
Neelma et al. [28]	55	55	N/A	N/A	N/A	N/A	N/A	N/A	N/A
Ugochukwu et al. [27]	52	7	6	2	0.170	0.031	0.176	-0.175	0.516
Farooq et al. [31]	23	23	2	1	-0.043	0.005	0.073	-0.186	0.099
Ashrad et al. [34]	40	54	5	1	-0.106	0.003	0.055	-0.215	0.002
Zardari et al. [36]	45	23	N/A	N/A	N/A	N/A	N/A	N/A	N/A
Mittal et al. [16]	30	30	0	0	N/A	N/A	N/A	N/A	N/A
Khan et al. [37]	75	75	N/A	N/A	N/A	N/A	N/A	N/A	N/A
Shah et al. [38]	32	32	5	3	-0.063	0.007	0.082	-0.224	0.099
Ullah et al. [39]	51	52	1	2	0.019	0.001	0.033	-0.046	0.084
Shah et al. [32]	200	200	22	34	0.060	0.001	0.035	-0.008	0.128
Cheema et al. [40]	50	50	3	8	0.100	0.004	0.062	-0.021	0.221
Waqar and Khan [1]	25	25	3	2	-0.040	0.007	0.085	-0.206	0.126
Beniwal et al. [41]	113	70	8	9	0.058	0.002	0.047	-0.034	0.149
Kapoor et al. [42]	25	25	0	0	N/A	N/A	N/A	N/A	N/A
Rathod et al. [35]	19	7	2	3	0.323	0.040	0.200	-0.068	0.715
Thakre et al. [43]	24	26	2	3	0.032	0.007	0.084	-0.133	0.197
Asif et al. [44]	40	40	0	0	N/A	N/A	N/A	N/A	N/A
Agrawal et al. [45]	25	22	3	4	0.062	0.011	0.105	-0.144	0.267

*Hospital Stay*

**Figure 7:**
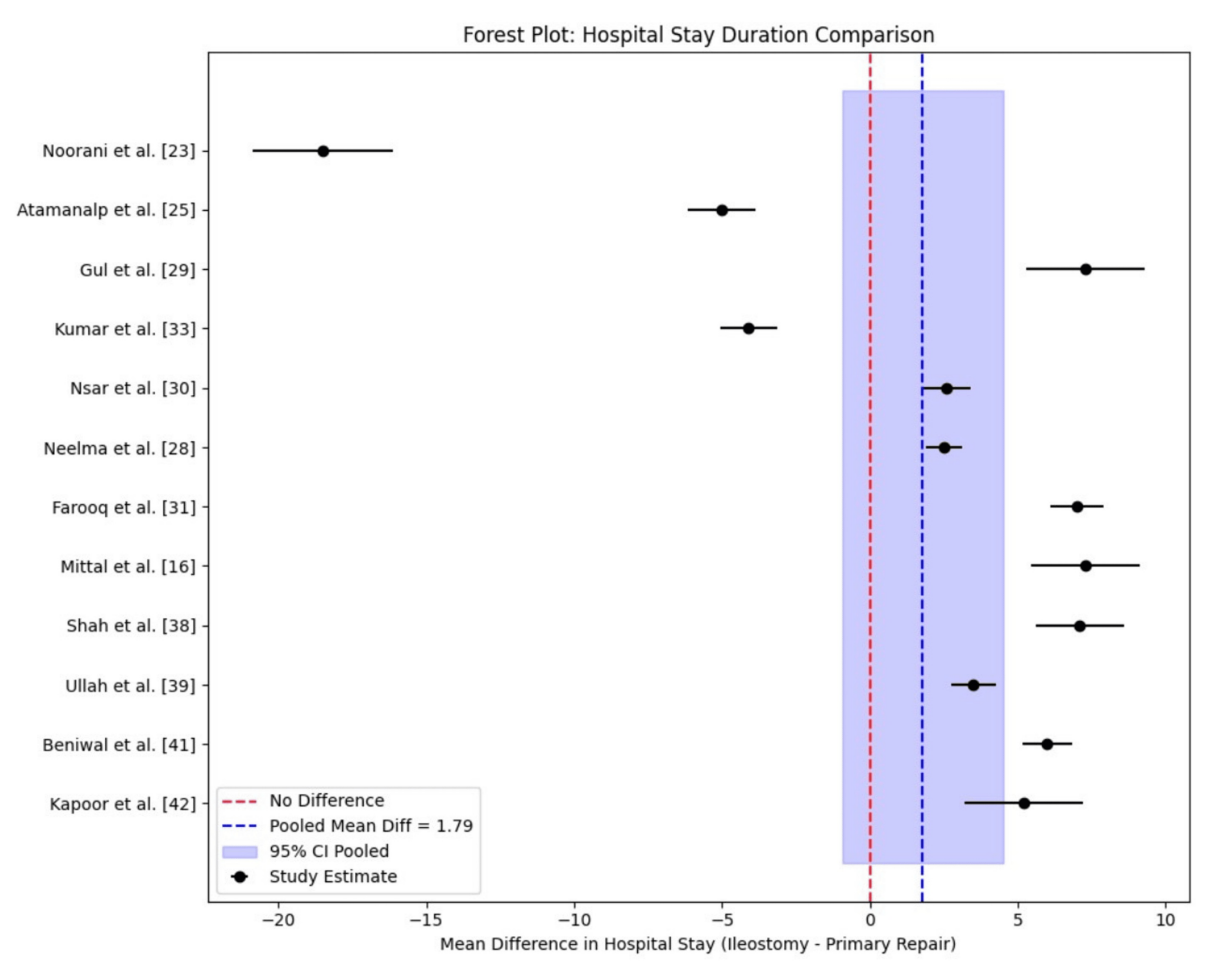
Forest plot of hospital stay duration

A meta-analysis of 13 studies revealed a mean difference in hospital stay duration of 1.79 days (ileostomy minus primary repair), with a 95% confidence interval of -0.82 to 4.40. This suggests ileostomy patients stay approximately 1.8 days longer than those undergoing primary repair, but the difference is not statistically significant, as the confidence interval includes zero (Table 4). The forest plot indicates substantial heterogeneity, with studies showing both shorter and longer hospital stays for each procedure (Figure 7).

**Table 4:** Hospital stay in primary repair vs. ileostomy CI: confidence interval

Author(s)	Intervention (primary repair)	Intervention (ileostomy)	Hospital stay days (primary repair)	Hospital stay day mean (primary repair)	Hospital stay days (ileostomy)	Hospital stay day mean (ileostomy)	Mean difference	Variance difference	Lower CI	Upper CI
Ali et al. [22]	20	30	N/A	N/A	N/A	N/A	N/A	N/A	N/A	N/A
Noorani et al. [23]	57	3	10-44	27	5-12	8.5	-18.5	1.47	-20.88	-16.12
Khan et al. [24]	40	40	N/A	N/A	N/A	N/A	N/A	N/A	N/A	N/A
Atamanalp et al. [25]	41	32	5-25	15	3-17	10	-5.0	0.34	-6.15	-3.85
Kouame et al. [4]	31	33	Shorter stay	N/A	Longer stay	N/A	N/A	N/A	N/A	N/A
Osifo and Ogiemwonyi [3]	2	2	N/A	N/A	N/A	N/A	N/A	N/A	N/A	N/A
Gul et al. [29]	52	21	14.23	14.2	21.53	21.5	7.3	1.04	5.31	9.29
Mishra et al. [26]	41	19	N/A	N/A	N/A	N/A	N/A	N/A	N/A	N/A
Kumar et al. [33]	42	58	13.66	13.7	9.62	9.6	-4.1	0.24	-5.06	-3.14
Nsar et al. [30]	30	30	6.5+/-1.1	6.5	9.1+/-2.4	9.1	2.6	0.17	1.80	3.40
Arshad et al. [46]	35	35	N/A	N/A	N/A	N/A	N/A	N/A	N/A	N/A
Neelma et al. [28]	55	55	6.78+/-2.1	6.8	9.29+/-2.88	9.3	2.5	0.10	1.89	3.11
Ugochukwu et al. [27]	52	7	N/A	N/A	N/A	N/A	N/A	N/A	N/A	N/A
Farooq et al. [31]	23	23	2-5	3.5	5-16	10.5	7.0	0.21	6.10	7.90
Ashrad et al. [34]	40	54	N/A	N/A	N/A	N/A	N/A	N/A	N/A	N/A
Zardari et al. [36]	45	23	Shorter stay	N/A	Longer stay	N/A	N/A	N/A	N/A	N/A
Mittal et al. [16]	30	30	14.23	14.2	21.53	21.5	7.3	0.89	5.46	9.14
Khan et al. [37]	75	75	Shorter stay	N/A	Longer stay	N/A	N/A	N/A	N/A	N/A
Shah et al. [38]	32	32	11.25+/-3.86	11.3	18.37+/-4.66	18.4	7.1	0.58	5.60	8.60
Ullah et al. [39]	51	52	7.8+/-2.3	7.8	11.3+/-3.1	11.3	3.5	0.15	2.75	4.25
Shah et al. [32]	200	200	N/A	N/A	N/A	N/A	N/A	N/A	N/A	N/A
Cheema et al. [40]	50	50	Shorter stay	N/A	Longer stay	N/A	N/A	N/A	N/A	N/A
Waqar and Khan [1]	25	25	N/A	N/A	N/A	N/A	N/A	N/A	N/A	N/A
Beniwal et al. [41]	113	70	10	10	16	16	6.0	0.18	5.16	6.84
Kapoor et al. [42]	25	25	15.4	15.4	20.62	20.6	5.2	1.06	3.18	7.22
Rathod et al. [35]	19	7	N/A	N/A	N/A	N/A	N/A	N/A	N/A	N/A
Thakre et al. [43]	24	26	N/A	N/A	N/A	N/A	N/A	N/A	N/A	N/A
Asif et al. [44]	40	40	Shorter stay	N/A	Longer stay	N/A	N/A	N/A	N/A	N/A
Agrawal et al. [45]	25	22	N/A	N/A	N/A	N/A	N/A	N/A	N/A	N/A

Discussion

The meta-analysis for morbidity indicates a statistically significant 18.4% higher complication rate with ileostomy compared to primary repair for typhoid intestinal perforation. This finding aligns with the broader literature, where primary repair is often favored in stable patients with limited contamination, as supported by Uba et al. [47], who reported a 15-20% morbidity advantage. Looking at the specific complications, wound infection emerges as a leading cause (23.5% in Ullah et al. [39] for primary repair and 16% in Shah et al. [32] for ileostomy), followed by sepsis (23.81% in Kumar et al. [33] for primary repair and 17.24% for ileostomy) and electrolyte imbalance (19.05% in Kumar et al. [33] for primary repair and 34.48% for ileostomy). These top three contributors underscore the physiological stress of ileostomy, particularly in managing fluid and electrolyte losses, a challenge also noted in a study by Clegg-Lamptey et al. [48] with rates up to 40% in resource-limited settings. Other significant complications include anastomotic leak (38.1% in Kumar et al. [33] for primary repair), fecal fistula ( 4.29% in Kumar et al. [33] for primary repair), and chest infection (33.3% in Noorani et al. [23] for ileostomy), reflecting the diverse postoperative risks. Notably, this review did not account for ileostomy-related complications such as stoma retraction, prolapse, or skin excoriation, which are reported at 5-10% incidence in other studies [49,50]. This omission may underestimate the true morbidity burden, as these complications often necessitate additional interventions and prolong recovery.

Regarding mortality, this review indicates a non-significant 3.1% higher mortality rate with ileostomy. Sepsis was identified as a dominant cause (66.7% in Atamanalp et al. [25] for ileostomy), followed by fecal fistula (22.2% in Atamanalp et al. [25]), with cardiopulmonary issues (11.1% in Atamanalp et al. [25]) and malnutrition ( 22% in Kouame et al. [4]) also significant. This pattern mirrors global trends, where delayed presentation and severe peritonitis elevate sepsis-related mortality to 30% or higher [51]. The wide range of RDs (e.g., -0.122 in Kumar et al. [33], favoring primary repair, to 0.323 in Rathod et al. [35], favoring ileostomy) suggests substantial heterogeneity, likely driven by patient selection bias. Ileostomy is often reserved for critically ill patients with multiple perforations or extensive contamination, a practice consistent with findings by Saxe and Cropsey [52], who noted higher mortality in diversion surgeries due to baseline risk rather than procedural failure. Conversely, primary repair's lower mortality in some studies (Ashrad et al. [34] (RD=-0.484)) may reflect its use in hemodynamically stable patients, though this advantage can be offset by anastomotic complications in under-resourced settings.

Hospital stay analysis reveals a pooled mean difference of 1.79 days, suggesting a non-significant 1.8-day longer stay with ileostomy. Individual study variations are striking: Gul et al. [29] and Mittal et al. [16] report a 7.3-day prolongation with ileostomy, while Atamanalp et al. [25] show a five-day advantage for primary repair. These differences may stem from postoperative complication management, with studies like Nsar et al. [30] (mean stay 9.1 days for ileostomy vs. 6.5 days for primary repair) suggesting that severe contamination drives longer stays. This review, however, did not consider reoperation rates for stoma-related issues, which can extend hospital stays by 5-7 days per procedure [53]. Comparative data from non-included studies, such as Contini [54] in Cameroon, report mean stays of 20-25 days with ileostomy due to limited intensive care, contrasting with the 10-15-day averages here, highlighting the role of healthcare infrastructure. Factors like delayed wound healing or recurrent sepsis, as seen in Kapoor et al. [42] (mean stay 20.6 days for ileostomy), further complicate stay duration, suggesting that local practices and patient comorbidities play critical roles. The observed heterogeneity across all outcomes reflects the multifactorial nature of typhoid perforation management. This variability is consistent with global data, where procedure choice is often dictated by clinical presentation, surgeon expertise, and resource availability rather than a universal superiority of one approach. 

## Conclusions

Primary repair demonstrates a significantly lower morbidity rate compared to ileostomy, primarily due to reduced occurrences of wound infection, sepsis, and electrolyte imbalance, though complications such as anastomotic leak and fecal fistula remain notable. Mortality differences between the procedures are not significant, with sepsis and fecal fistula being the primary contributors, likely influenced more by patient condition than the choice of procedure. Hospital stays are slightly longer with ileostomy, though the difference is not significant, with variations suggesting dependence on context-specific factors. These findings highlight the need to tailor surgical approaches to patient stability and available resources. Future research should incorporate ileostomy-related complications, reoperation rates, and standardized data collection to refine outcome comparisons and enhance evidence-based guidelines for typhoid perforation management.

## Appendices

**Table 5:** Search strategy MeSH: Medical Subject Headings

Database	Search	Papers
PubMed	("Typhoid Fever"[Mesh] OR "Intestinal Perforation"[Mesh]) AND ("Surgical Procedures, Operative"[Mesh] OR "Ileostomy"[Mesh]) AND ("Morbidity"[Mesh] OR "Mortality"[Mesh] OR "Length of Stay"[Mesh]) AND ("primary repair" OR "simple closure" OR "loop ileostomy")	1,624
Google Scholar	"typhoid ileal perforation" "primary repair" "loop ileostomy" "morbidity" "mortality" "hospital stay"	500
Cochrane Library	[ti,ab] ("typhoid ileal perforation" OR "typhoid intestinal perforation") AND [ti,ab] ("primary repair" OR "simple closure") AND [ti,ab] ("loop ileostomy" OR "ileostomy") AND [ti,ab] ("morbidity" OR "mortality" OR "hospital stay")	12
African Journals Online	"typhoid ileal perforation" "primary repair" "loop ileostomy" "morbidity" "mortality" "hospital stay"	21
